# Maintaining Economic Value of Ecosystem Services Whilst Reducing Environmental Cost: A Way to Achieve Freshwater Restoration in China

**DOI:** 10.1371/journal.pone.0120298

**Published:** 2015-03-24

**Authors:** Mingli Lin, Zhongjie Li, Jiashou Liu, Rodolphe E. Gozlan, Sovan Lek, Tanglin Zhang, Shaowen Ye, Wei Li, Jing Yuan

**Affiliations:** 1 State Key Laboratory of Freshwater Ecology and Biotechnology, Institute of Hydrobiology, Chinese Academy of Sciences, Wuhan, China; 2 Sanya Institute of Deep-sea Science and Engineering, Chinese Academy of Sciences, Sanya, China; 3 UMR207 IRD, CNRS 7208-MNHN,-UPMC, Muséum National d’Histoire Naturelle, Paris, France; 4 UMR 5174 EDB, CNRS-University Paul Sabatier, 118 route de Narbonne, Toulouse, France; Bournemouth University, UNITED KINGDOM

## Abstract

Freshwater fisheries are central to food security in China and this remains one of the most important priorities for the growing human population. Thus, combining ecosystem restoration with economics is pivotal in setting successful conservation in China. Here, we have developed a practical management model that combines fishery improvement with conservation. For six years, a ban on fertilizer and a reduction of planktivorous fish stocking along with the introduction of both mandarin fish *Siniperca chuatsi* and Chinese mitten crab *Eriocheir sinensis* was apparent in Wuhu Lake, a highly eutrophic lake located in the middle reaches of the Yangtze River. Annual fish yield decreased slightly after the change in management, whereas fisheries income increased 2.6 times. Mandarin fish and Chinese mitten crab accounted for only 16% of total fisheries production but for 48% of total fisheries income. During this six year period, water clarity increased significantly from 61 cm to 111 cm. Total nitrogen, total phosphorus and chlorophyll decreased significantly from 1.14 to 0.84 mg/L, 0.077 to 0.045 mg/L, and 21.45 to 11.59 μg/L respectively, and macrophyte coverage increased by about 30%. Our results showed that the ecological status of shallow lakes could be rapidly reversed from eutrophic to oligotrophic using simple biomanipulation, whilst maintaining fisheries economic value. It also offers a better approach to shallow fisheries lake management in Asia where traditionally the stocking of Chinese carp and use of fertilizers is still popular.

## Introduction

China has now become the most significant contributor to world aquaculture with an annual production of 41 million tonnes (i.e. 62 percent of global aquaculture production in 2012) [1]. However this rapid economic development has also led to a range of environmental changes directly impacting fisheries [2]. For example, lake aquaculture (i.e. 998 000 ha) represents the main part of inland fisheries in China [3] and with traditional techniques that include application of mixed fertilizers (ammonium, phosphate and nitrate based) along with intensive stocking of carp (e.g. grass carp *Ctenopharyngodon idellus*, planktivorous silver carp *Hypophthalmichthys molitrix* and bighead carp *Aristichthys nobilis*) [4] has caused a significant decrease in macrophytes [5,6], increased nutrient load and reduced water quality [7]. Due to the rapid recent eutrophication of Yangtze River floodplain lakes [8], a set of sustainable management strategies that combine economic sustainability of fisheries and the conservation of freshwater ecosystems is necessary [9].

It is known that the specific and functional diversity of aquatic ecosystems, in particular lotic ones, is regulated by a set of “bottom-up” and “top-down” processes [10,11]. Thus, the current use of fertilizers in these shallow lakes lead to a bottom up eutrophication of these ecosystem via an increase of nutriment load alongside a bottom up impact on macrophytes and plankton via the choice of fish species stocked [10,11]. For example, carp species typically forage on benthos and they put many nutrients back into the water column. This, along with increased nutrient load via the production of faeces, will sustain planktonic algal blooms and reduce the community of aquatic macrophytes, limiting their access to nutrients in the substrate and reducing light level in the water column. Thus, changing community structure through the introduction of top predators with less impact on nutrient load coupled with a reduction in fertilizer and carp species could lead to a whole food web trophic cascade and result in improved water quality and increased macrophyte abundance [12]. However, a knowledge gap remains regarding the long-term economic and ecological sustainability of using this type of bio-chemical manipulation as a tool for lake restoration [13–16]. There are few examples of lake restoration via bio-manipulation processes [14,15,17], mostly in North American and European Lakes but that combine both ecosystem restoration with fisheries economics. The added challenge within an East Asian context is to maintain the economic value of the fisheries in addition to the lake restoration, as this aspect is central to the local population endorsing any new management options. In effect, previous research in shallow lakes of the Yangtze floodplain only focused on estimating the stocking capacity of piscivorous fish, the biomass and production of small fish as well as bioenergetics of mandarin [18–24].

Here using an un-replicated large-scale long-term experiment on an existing eutrophic shallow lake, we tested the effect of nutrient control and change in community composition (i.e. fish, crustaceans) on the ecological status of the lake and the economics of the associated fisheries. Specifically, we tested if 1) using simple rules arising from our current theoretical understanding of “bottom-up” and “top-down” processes, we could rapidly improve the water quality and macrophyte abundance of shallow lakes and 2) we could achieve this improved ecological status without impacting the economic value of the fisheries based on the use of replacement species of high market value. [18–20]

## Materials and Methods

### Ethics statement

The studied lake belongs to the Chinese government and is rented to the private Wuhu Lake Fishery Management Company for management (188# Dazui Village, Liuzhi Street, Huangpi District, Wuhu City, Hubei Province, China). This is the company, which has given us the permission of water quality sampling and given us the fishery management data. We confirm that no fish were caught for the purposes of research.

### Study area

**Fig 1:**
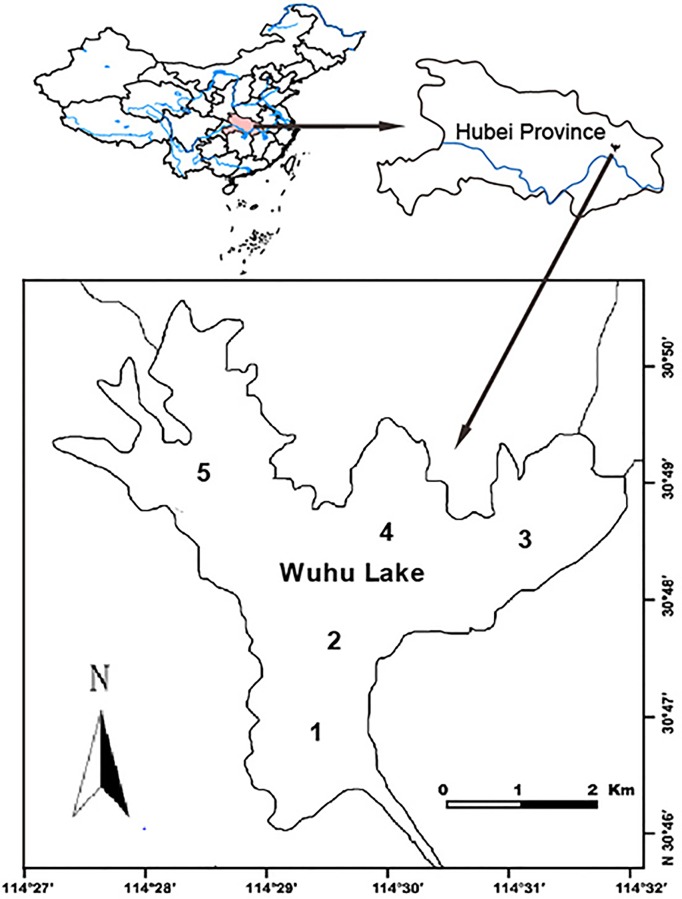
Location of Wuhu Lake located in the middle reaches of the Yangtze River, Hubei Province, China. Sample sites are displayed as number on the map.

The study was carried out between 2006 and 2011 in Wuhu Lake located in the middle reaches of the Yangtze River, Hubei Province, China (Fig 1). This is a very typical large (i.e. 2133 ha) but shallow lake (mean depth 2.6 m) disconnected by a sluice gate from the Yangtze River in 1952. It originally hosted a wide diversity of submersed macrophytes (mainly *Ceratophyllum demersum*, *Vallisneria spiralis*, *Trapa bispinosa* and *Potamogeton wrightii*), the baseline biomass was 3800g/m^2^ [25], but almost disappeared when traditional fisheries started post 1995. By contrast the density of phytoplankton increased from 89.3×10^4^ind/L in 1995 to 1726×10^4^ind/L in 2005 [25]. Since 1995, the lake was managed to culture domesticated carp species including *C*. *idellus*, *H*. *molitrix*, and *A*. *nobilis* at stocking densities of 20–55 kg/ha, with the additional production of mandarin fish *Siniperca chuatsi*. To support the fish culture, plankton biomass was enhanced by the addition of non-organic fertilizers (417 kg/ha ammonium bicarbonate and superphosphate annually during 2002 to 2006) and organic fertilizers (1327 kg/ha brewer's grains and animal faeces annually during 2005–2007).

### Biomanipulation

**Fig 2:**
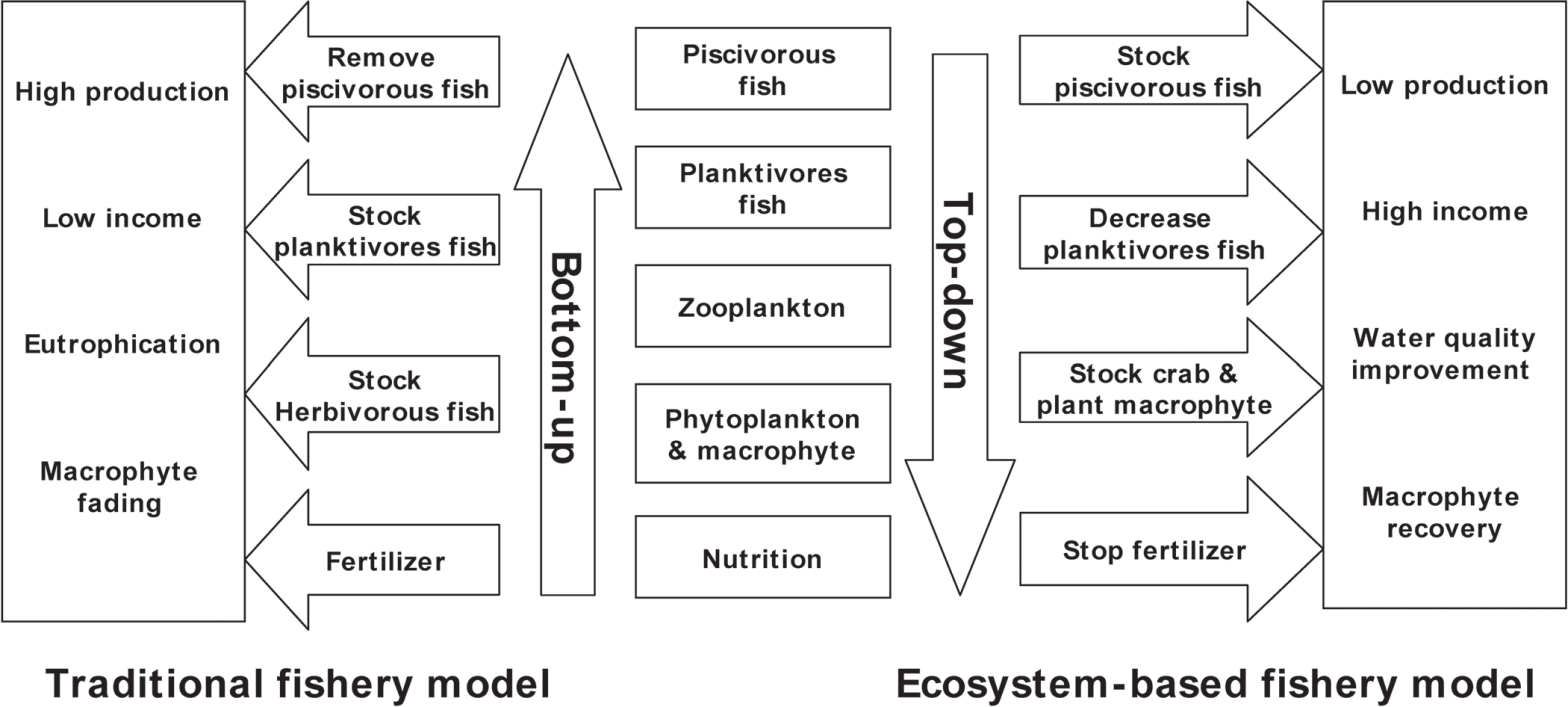
Traditional fishery model and ecosystem-based fishery model in Yangtze lake.

At the beginning of each year an agreed biomass per species (fish and crab) is stocked and fertiliser added. At the end of the year, all species are harvested and sold. Each year is thus a replicated experiment with pre-set species composition and biomass. Here, two replications (2006–07) consist of ‘classic’ stocking of carp and fertiliser (pre biomanipulation), followed by four replications post biomanipulation (2008–11). In 2008, we started a combined top-down (1) and bottom up (2) bio-manipulation of the lake by 1) stopping the stocking of *C*. *idellus*, decreasing the biomass of *H*. *molitrix*, *A*. *nobilis* from 8.9 ± 0.9 × 10^4^ kg to 5.6 ± 1.5 × 10^4^ kg and introducing the Chinese mitten crab *Eriocheir sinensis* a species of high economic value (Table 1); 2) stopping the fertilisation of the lake (i.e. no fertilisers or brewer’s grain) and replanting *Vallisneria spiralis* to recover submerged macrophytes to provide refuge and food for the Chinese mitten crabs. The stocking biomass of the coin-sized mitten crab (i.e. ~ 5 g/ind.) was 1.58 ± 0.05 ×10^4^ kg and approximately 20 ± 5.3 × 10^4^ mandarin fingerlings (~ 3 cm total length). Both stocking densities followed recommendations from [23,26]. The traditional fishery model and our ecosystem-based model are presented as Fig 2.

**Table 1:** Stocking number and biomass of carp, Mandarin fish and Chinese mitten crab pre (2006–2007) and post bio-manipulation (2008–2011) of Lake Wuhu.

Year	Silver carp and bighead carp	Mandarin fish	Chinese mitten crab
	10^4^kg	10^4^ind	10^4^kg
2006	8	4.8	0
2007	9.8	4	0
2008	3.6	25	1.7
2009	9.8	4	1.5
2010	3.6	25	1.6
2011	5.2	26	1.5

### Sample collection and analysis

Wuhu Lake Fishery Management Company is a partnership company, and the sale data of different fish species were calculated precisely as they related to the profit share. Therefore, catch data of the crab and fish from 2006 to 2011 were directly obtained from sale records. Water quality was measured during May and October during 2006 to 2011 from five constant sampling sites in the lake (Fig 1). Water transparency (SD) was measured by Secchi Disc. In order to measure the content of total nitrogen (TN), total nitrogen (TN) and chlorophyll a (Chl a), 5 L water were sampled in each site and then transported to the laboratory. TN, TP and Chl a content was quantified immediately by Alkaline potassium persulfate digestion-UV spectrophotometric, Ammonium molybdate spectrophotometric and the homochromy spectrophotometric methods respectively [27]. All measuring procedures strictly followed the protocol of Chinese national standards [28] and finished within 24 h after sampling to avoid error cause by water quality changing.

In order to determine the coverage rate of submersed macrophytes, the whole lake was divided evenly into several polylines and then 48 sites were randomly selected above these lines. All these sites were recorded by GPS and remained fixed for the length of the study. Submersed macrophytes were sampled by scythes (1/5 m^2^) 2–3 times at each site just above the sediment. After scything, plants were gathered with a 425 μm handnet and put into plastic bags. In the laboratory, the plants were rinsed, rid of superfluous water and wet weight taken. The rate of macrophyte coverage was calculated as an occurence ratio across all 48 sampling sites.

Mean and standard error (s.e.) were calculated and the environment data testing for normality used a Shapiro-Wilk test. Independent Sample T-test (parametric) and Mann-Whitney-Wilcoxon Test (non-parametric) tested the significance of the differences in water quality before and after the start of the bio-manipulation. All analyses were performed with R [29].

### Fishery economic value

In this study, two factors determined the economic value of the fishery. One was the yield and the other one was the market price ($.kg^-1^) for each individual fish species. As the market price changed each year, the economic value did not necessarily follow the yield data.

## Results

### Environmental change

**Fig 3:**
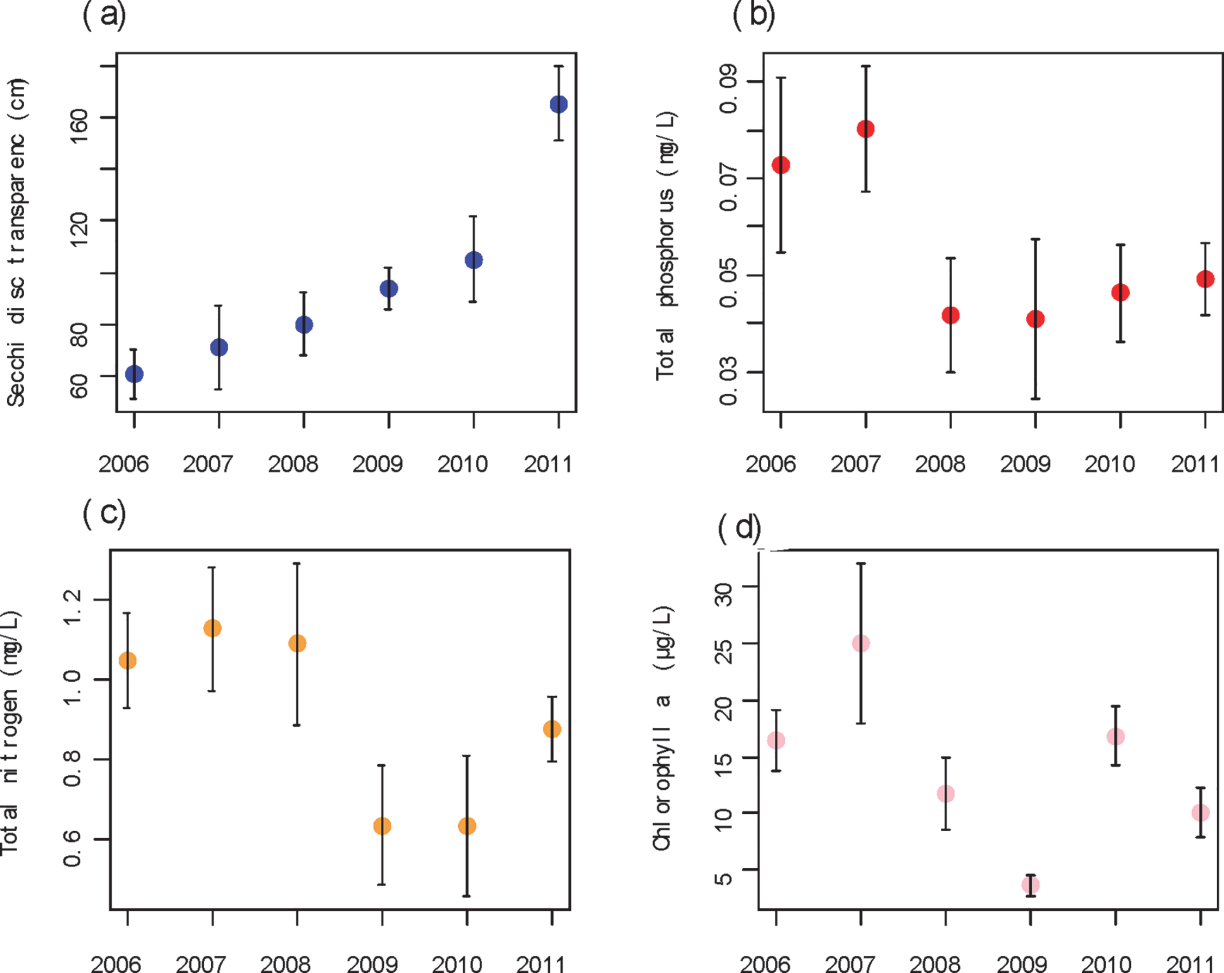
Water quality parameters of Wuhu Lake (China) before and after the start of bio-manipulation in 2008.

Following the change in the fishery management in 2008, there was a significant increase in water clarity and overall quality (*P* < 0.05; Table 2, Fig 3). Macrophyte coverage also increased with almost no submersed macrophytes observed in 2006 to 31% submersed macrophyte (mainly *V*. *spiralis*) coverage by summer 2010. Annual macrophyte cover was only estimated pre and post biomanipulation treatment to avoid disturbance of the macrophyte recolonisation process.

**Table 2:** Mean water parameters and standard error pre (2006–2007) and post bio-manipulation (2008–2011) of Lake Wuhu.

Water parameters	Pre bio- manipulation		Post bio- manipulation		*p* value	Test	df (W)
	Mean	SE	Mean	SE			
Water clarity (cm)	65.8	9	111.2	9.6	0.002	t	26
Total phosphorus (mg/L)	0.077	0.01	0.045	0.005	0.038	MWW	(400)
Total nitrogen (mg/L)	1.14	0.11	0.84	0.07	0.004	MWW	(361)
Chlorophyl a (μg/L)	21.45	4.5	11.59	1.46	0.025	MWW	(369)

Statistical test (t = t-test; MWW = Mann-Whitney-Wilcoxon test), significance (*P*) and degree of freedom (df) are included.

### Fishery catch

**Fig 4:**
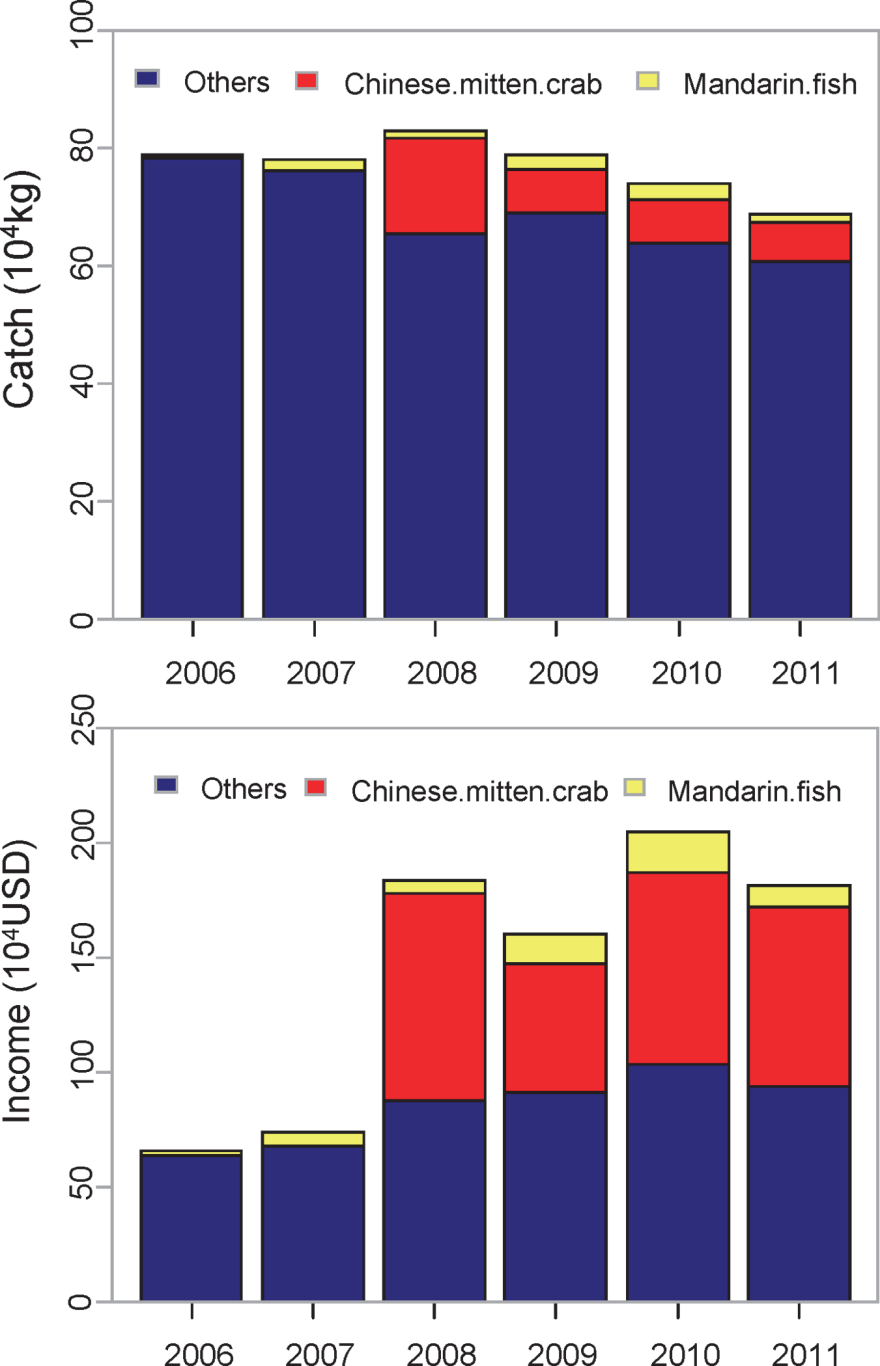
Comparison of fisheries catch and income between pre bio-manipulation and post bio-manipulation in Wuhu Lake (2008 was the start of the change in the management practice). Price of Chinese mitten crab has doubled between 2008 and 2010.

Within four years following the start of the management change, the overall annual fish yield decreased by 3% from pre biomanpulation mean ± s.e. = 785000 ± 5000 kg to post biomanpulation 763000 ± 30000 kg; the Chinese carp, including *H*. *molitrix*, *A*. *nobilis C*. *idellus* and *Cyprinus carpio* decreased from mean ± s.e. = 630400 ± 67000 kg to 477625 ± 161640 kg. Following the start of the management change, the total of mandarin fish and Chinese mitten crab established a sustainable annual crop at mean ± s.e. = 20400 ± 3706 kg and 93600 ± 22124 kg respectively (Fig 4) and the overall yield of mandarin fish and Chinese mitten crab accounted for about 15% of the total fisheries production.

### Economic value

The fisheries income increased significantly following the bio-manipulation (Fig 4), with an annual average income of about $1.83 million USD, 2.6 times that of 2006–2007 (i.e. ~$0.70 million). During this period, mandarin fish and Chinese mitten crab alone accounted for about mean ± se = 48% ± 2 of the total income generated.

## Discussion

Within four years, coinciding with the change in management, the transparency of Wuhu Lake water has more than doubled, the macrophyte biomass has increased by thirty percent and the economic value of the fishery has tripled. The main aspect underpinned by these results is that in a Chinese context, improving ecological status of lakes does not have to be achieved to the detriment of ecosystem service sustainability. On the contrary, it is the consideration of the economic dimension that will allow the long-term sustainability of biodiversity and ecological restoration. Lake Wuhu is very typical of thousands of lakes on the floodplain of the Yangtze River that were converted into fisheries in 1980s [30]. Animal protein and food security has been and remains one of the most important priorities for the growing population of China and innovation in environmental management is central to achieving that subtle balance between conservation and economics.

In aquatic ecosystems, the flux of energy via predator-prey interaction is generally directed from small-sized, abundant organisms to rare, larger bodied species [31] with a high turnover at the lower levels of a foodweb, whereas larger animals fix nutrients for longer time periods, making them unavailable. However in these complex foodweb structures the presence of keystone species [32], such as carp species [33], exerts a disproportionate influence on the pattern of species diversity in a community, in particular if they are stocked at high densities, removing macrophytes and lowering water clarity [5, 6]. Until now, stocking *H*. *molitrix* and *A*. *nobilis* along with lake fertilization has been the dominant management model of shallow lakes fisheries in the middle and lower reaches of the Yangtze River, allowing stable yield and low-risk profit but leading to nutrient enrichment of the lakes and associated problems of eutrophication [8]. This study goes further than previous research on these shallow lake fisheries of the Yangtze [18–24] to show that a careful selection of high economic value species can achieve high economic value while improving the water quality in a traditional fishery lake. As the application of fertilizers along with intensive stocking of carp are still popular in China, this study is pivotal in demonstrating to local stakeholders as well as policy makers that in a freshwater fisheries context the economic and environmental aspects are not necessarily antagonistic.

This bio-manipulation indicates that with a slight change in the composition of top predators and stopping using fertilizers, sustained by a reduction of carp production that limits the bio-engineering effect of fine particle re-suspension in the water column, macrophytes can establish large biomass nitrogen levels decrease. Nutrient control, especially TP load, restricts phytoplankton growth and thus helps water clarity [14,15,17,34], whilst apex predators preying on zooplanktivorous species allowed zooplankton growth and therefore increased phytoplankton grazing also leading to an increase in water clarity [16,35]. Similar effects were observed in the Great Lakes after the accidental introduction of another ecosystem engineer the zebra mussel *Dreissena polymorpha* [36], and in Lake Mendota after piscivore stocking [37]. Based on previous research [12], we speculated that mandarin fish triggered a so-called “trophic cascade” whilst the removal of grass carp and end of the fertilization process triggered a bottom-up effect [38]. The two effects are not contradictory and both possibly leading to synergetic effects such as the reduction of eutrophication. A traditional measure for a trophic cascade is a change in plant biomass [39], which can be taken as a measure of productivity. Here, despite not having a direct measure of the annual re-growth of the macrophyte community, the overall biomass of the macrophytes in Lake Wuhu pre and post bio-manipuation increased markedly.

However, the stocking balance needs to be carefully weighed as over stocking of Chinese mitten crab could lower the abundance of macrophytes and negatively impact the fisheries yield and thus income [26]. In light of this, we suggest that the stocking density used in this bio-manipulation experiment corresponds to the upper stocking limit. Although we will never restore the integrity of Wuhu Lake’s ecosystem as it will always be managed due to socio-economic forces, it is still important to integrate the dimension of resilience in the future management of these lakes [40]. Resource based systems like fisheries should be kept in a stable state that guarantees optimal exploitation, also known as imposed resiliency [41]. However, stable systems do not have to be resilient and a higher biodiversity, although artificially driven, will be a key driver of resilience in these shallow lakes in future years.
